# Physio-metabolic response, immune function, epigenetic markers, and reproductive performance of rabbits under environmental stress: the mitigating role of boswellia essential oil nanoemulsion

**DOI:** 10.1186/s12917-025-04587-1

**Published:** 2025-03-15

**Authors:** Sameh A. Abdelnour, Mahmoud Abdelaal, Ramya Ahmad Sindi, Mohammed A. Alfattah, Wael A. Khalil, Laila B. Bahgat, Asmaa M. Sheiha

**Affiliations:** 1 Department of Animal Production, Faculty of Agriculture, Zagazig University, Zagazig, 44511 Egypt; 2 Department of Clinical Laboratory Sciences, Faculty of Applied Medical Sciences, Umm Al-Qura University, Makkah, Saudi Arabia; 3 Department of Biology, College of Science, Jazan University, PO Box 114, 45142 Jazan, Saudi Arabia; 4 Department of Animal Production, Faculty of Agriculture, Mansoura University, Mansoura, 35516 Egypt

**Keywords:** Rabbit, Ferroptosis, Reproductive, Heat stress

## Abstract

Global warming poses a significant threat to reproductive health of rabbits. Sustainable nutritional strategies are crucial for ensuring rabbit production and maintaining food security under these challenging conditions. This study sought to assess the protective benefits of dietary boswellia essential oil nano-emulsion (BEON) against oxidative stress, immune dysregulation, ferroptosis, and organ damage in female rabbits exposed to severe thermal stress. A total of 120 female rabbits were divided into four groups of 30 rabbits each. The rabbits were fed a basal diet supplemented with 0 (BEON0), 0.25 (BEON0.25), 0.5 (BEON0.5), and 1.0 (BEON1.0) mL of BEON per kilogram of diet. Results demonstrated that the BEON1.0 group exhibited significantly higher levels of IgG, superoxide dismutase (SOD), and glutathione peroxidase (GPx), while the BEON0.25 group showed elevated levels of IgM, catalase, and total antioxidant capacity (TAC) (*P* < 0.05). All BEON treatments significantly reduced malondialdehyde (MDA) levels (*P* < 0.01). Serum levels of progesterone, luteinizing hormone (LH), and follicle-stimulating hormone (FSH) were significantly elevated in the BEON0.5 and BEON1.0 groups compared to the control group (*P* < 0.01). A significant decrease in adipokine levels was observed in all BEON-supplemented groups compared to the control group (*P* < 0.05). All BEON groups demonstrated a modulation of ferroptosis pathways, characterized by decreased heat shock protein 70 (*HSP70*) expression and upregulated expression of glutathione peroxidase 4 (*GPX4*) and cystine transporter solute carrier 7A11 (*SLC7A11*) in ovarian tissues (*P* < 0.01). Furthermore, DNA methyltransferase 1 (*DNMT1*) expression increased in a dose-dependent manner with increasing BEON supplementation. Histological analysis revealed an improvement in the architecture of the liver, uterine horns, and ovarian tissues in rabbits fed BEON. Integrating BEON at doses of 0.5–1.0 mL/kg diet significantly improved reproductive performance in stressed female rabbits. PCA and correlation analyses demonstrated a positive correlation between BEON supplementation and immune function, reproductive hormone levels, and antioxidant status, while a negative correlation was observed with MDA and adipokine concentrations in rabbit serum. In conclusion, BEON supplementation demonstrates promise as a sustainable nutritional strategy for the rabbit industry, particularly in mitigating the challenges posed by global warming.

## Introduction

Climate change is driving a significant increase in seasonal temperatures and a concomitant increase in the frequency and severity of heat extremes. These changes pose a threat to global food sources, with the livestock industry being particularly vulnerable. Rabbits, as an important source of animal protein, play a crucial role in global food production and significantly contribute to human nutritional needs [1]. Due to the low number of sweat glands in rabbits, they are vulnerable to high environmental temperatures, which can significantly impact their food supplies [2, 3]. Heat stress (HS) has a significant impact on physiological health by disrupting hormonal synthesis and causing immune dysfunction, leading to an overall state of physiological imbalance [4–7]. HS also destroys the antioxidant profile, promotes lipid peroxidation, and induces ferroptosis [5, 8, 9]. This imbalanced state leads to reduced feed intake and feed efficiency, resulting in a decline in ovarian activity [10], ultimately inducing infertility under HS conditions in rabbits [4, 11]. Genetically, HS can reduce the expression of antioxidant genes such as Nrf2, HO-1, NQO1, and GPX1, while the addition of certain phytochemicals can modulate these alterations in rabbits [12]. Epigenetics plays a pivotal role in biological processes, such as heat adaptability, growth, and gene expression regulation. Rabbit adaptability to HS is significantly influenced by epigenetic modifications, such as histone modifications and DNA methylation [13]. Evidence suggests that these epigenetic mechanisms, in conjunction with genetic factors, play a crucial role in regulating responses to HS. A recent study demonstrated that HS can induce epigenetic alterations in rabbits by decreasing DNA methylation levels [13]. Previous research has shown that certain herbal drugs can modulate DNA methylation patterns in livestock [14]. Thereafter, it’s critical to find a novel sustainable nutritional intervention for rabbit production to mitigate the challenges posed by global warming.

In recent years, nanotechnology has emerged as a promising frontier in the livestock industry, offering innovative solutions to challenges in food production, animal health, and environmental sustainability [15]. Herbal extracts and essential oils have been widely used to enhance animal growth, health, and well-being [7, 16]. Due to their bioactivity and storage stability, essential oils have been explored as potential alternatives to antibiotics in animal and veterinary sciences [17]. However, their widespread use in the animal industry is hindered by limitations such as poor stability, low bioavailability, and solubility constraints [7, 16]. Nano-emulsions offer a potential solution to these challenges by enhancing bioactivity, improving storage stability, and increasing the bioavailability and gut absorption of essential oils [7].

*Boswellia carterii,* commonly known as frankincense, is a moderate to large-sized, branching tree native to the dry mountainous regions of North Africa, India, and the Middle East. *Boswellia carterii* oil (BEO) has a long history of use in traditional medicine and aromatherapy. Notably, it possesses antimicrobial properties, finding applications in both medicine and the food industry. The incorporation of Boswellia raw material into animal diets has shown promising results, including improved growth, enhanced antioxidant properties [18, 19], and strengthened immunity [20]. Furthermore, it has been shown to mitigate metabolic disorders [21], and contribute to overall animal health and well-being. The primary constituents of BEO include α-pinene (37.0%), α-limonene (19.8%) [22], and p-cymene (6.3%) [23] and along with a significant proportion of boswellic acids (25–30%) (25–30%) [24]. These bioactive molecules contribute to the robust antioxidant, antimicrobial [25] anticancer [26], anti-apoptosis [27], and anti-inflammatory effects of BEO. Furthermore, α-Phellandrene, another component of BEO, has been shown to promote vascular endothelial growth factor [28], which may contribute to improved heat dissipation. In a study on mice, BEO demonstrated the capacity to ameliorate reproductive disorders associated with obesity by modulating lipid profiles, enhancing immune function, and mitigating apoptosis [29]. Given the documented biological activities of BEO, we hypothesized that a novel nano-emulsion formulation of this essential oil would exhibit enhanced efficacy in mitigating the negative impacts of heat stress (HS) in female rabbits. Therefore, this study was designed to investigate the protective role of boswellia essential oil nano-emulsion (BEON) on redox status, immune function, hormone levels, adipokines concentrations, organ histology, and reproductive performance in female rabbits subjected to heat stress.

## Materials and methods

The present experiment was conducted at Rabbit Farms, Agricultural San Elhajr Company, San Elhajr, Sharkia (30°58′37″N 31°52′48″E) in accordance with the ethical guidelines of the Scientific Research Ethics Committee at Zagazig University. The experiment was carried out during the hot summer season from May to October 2023.

### Ethical statement

All procedures and experimental protocols were conducted in strict accordance with Directive 2010/63/EU of the European Parliament and of the Council of 22 September 2010 on the protection of animals used for scientific purposes. The experimental procedures were approved by the Zagazig University Scientific Research Ethics Committee (Ethical code: ZU-IACUC/2/F/174/2022) and conducted in accordance with the ARRIVE Guidelines 2.0 [30, 31].

### Preparation of boswellia essential oil nano-emulsion (BEON)

The *boswellia carterii* oil (BEO) was obtained from AB Chem Company (Mansoura, Egypt) for the preparation of nano-emulsion boswellia essential oil (BEON). A single layer of BEON oil-in-water nano-emulsion was designed by [7]. To prepare the nano-emulsion, 2.5 mL of BEO was combined with 1 mL of a surfactant solution (Tween 80, 5–6% v/v). Subsequently, 10 mL of water was added dropwise under constant magnetic stirring at 25°C. The water was added at a rate of approximately 1.0 mL/min. Following this, the emulsion underwent sonication for 30 min in an ultrasonic bath (Sonix, USA). Subsequently, it was further homogenized using an ultrasonic probe (Model CV 334) attached to a homogenizer (Sonics Vibra-cell™, Model VC 505, Inc., USA). This process was repeated several times to determine the volume of the BEON for this study. The homogenization process was conducted at an amplitude of 60% for 5 min with a pulsing cycle of 1 s ON and 1 s OFF. The dose of BEON was incorporated into the diets following the experimental protocol outlined in [7].

### Physicochemical features of BEON

**Fig. 1:**
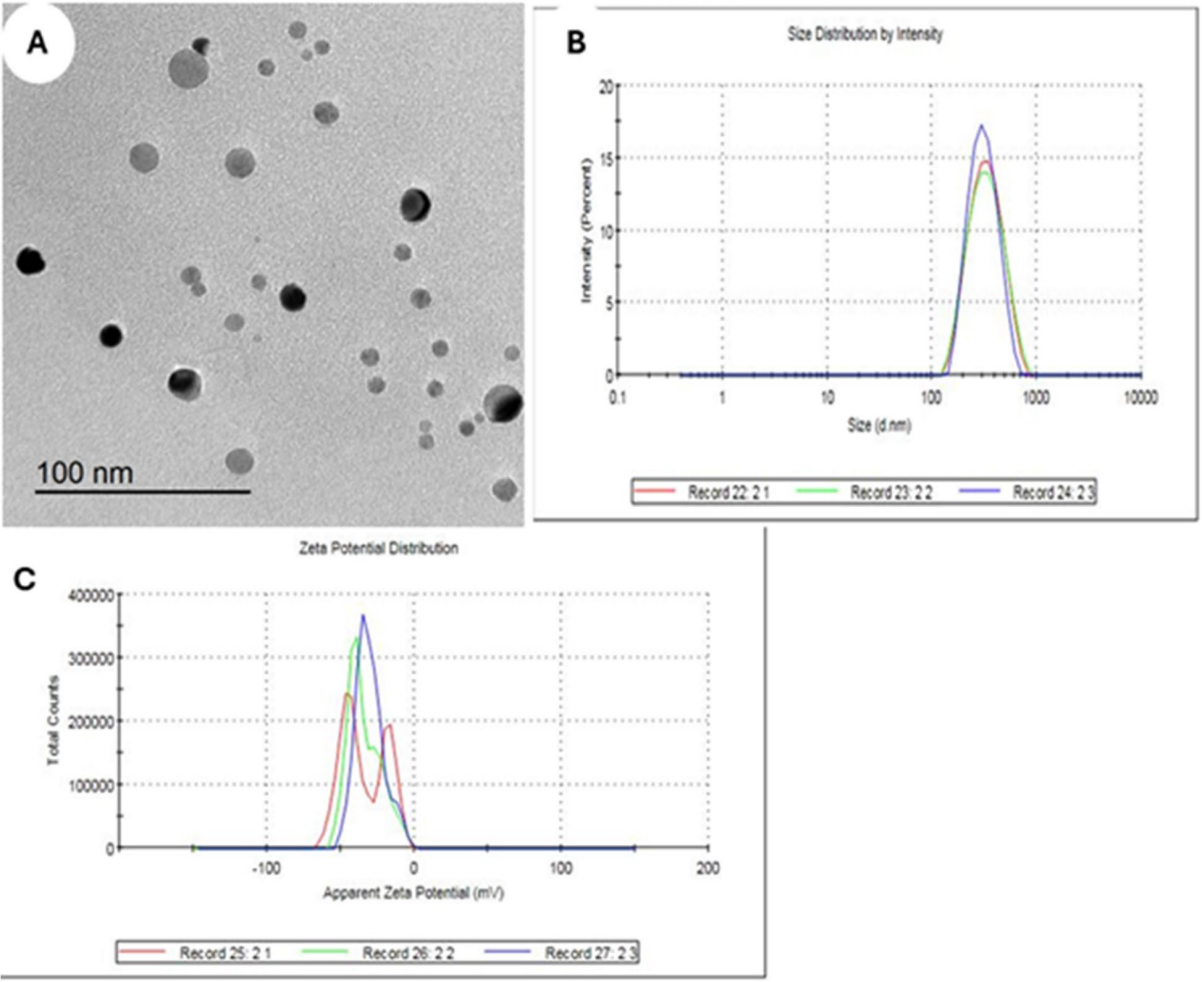
**A-C** The characterization of Boswellia Essential Oil Nanoemulsion (BEON) was used in this experiment. The TEM indicated the BEON droplets were spherical (**A**). The mean droplet diameter was 299.8 nm (**B**). Polydispersity index (PDI) was 0.166, indicating better stability (**C**)

The morphology of freshly synthesized BEON was characterized using transmission electron microscope (TEM; JEOL JEM-2100, Tokyo, Japan) at 160 kV. Digital Micrograph and Soft Imaging Viewer software were employed for image acquisition and analysis. The surface charge of the nano-emulsion particles (Z-potential) represented by the zeta potential, was determined using a Zetasizer Nano ZS analyzer (Fig. 1).

### Meteorological parameters

During this trial, ambient temperature and relative humidity values were recorded to determine the severity of HS on rabbits based on the Temperature Humidity Index (THI). The equation for THI was calculated according to the equation mentioned in Marai et al. [3]. THI = dp- [(0.31–0.31 (RH/100)) × (dp-14.4)], where Tdb is the dry bulb temperature in degrees Celsius and RH is the relative humidity as a percentage. The calculated THI values were then categorized as follows; > 30.0 (very severe HS), 29.0 to 30.0 (severe HS), 27.8 to 28.9 (moderate HS) and < 27.8 (absence of HS).

### Animals, experimental design and diets

A total of 120 healthy mature New Zealand White rabbit does, weighing an average of 2.98 kg ± 40.27 g and six months of age, were included in this trial. The female rabbits were individually housed in galvanized wire cages with standard dimensions (60 × 55 × 40 cm^3^). Each cage was equipped with a kindling nest-box (43 × 26 × 26 cm^3^). The rabbit cages were supplied with manual feeders and an automatic system of nipple drinkers to provide fresh water ad libitum [4]. Female rabbits received a basal diet supplemented with *boswellia* essential oil nano-emulsion (BEON) at four levels: 0 (control), 0.25, 0.5, and 1.0 mL BEON per kilogram of diet [7]. This feeding regimen was maintained for six months during the Egyptian summer. During diet formulation, the doses of BEON at levels (0.25, 0.5 and 1 mL) were added according to the study protocol. The experiment was conducted over four parities. Does were presented to bucks 12–14 days postpartum, coinciding with the optimal time for mating receptivity in this breed [4]. Additionally, the bucks were proven for their fertility based on the farm records. The dietary component and chemical composition of the diet used in this experiment are presented in Table 1. The diet, formulated to meet the nutrient requirements during pregnancy and lactation [32], contained 18.2% crude protein and 2650 kcal/kg digestible energy. Does were maintained under hygienic, managerial, and sanitary conditions. Throughout the experimental period, animals were subjected to regular examinations to monitor their body condition and health status.

**Table 1:** The dietary component and chemical composition of the diet used in this experiment

Ingredients (%)	
Barley grain	14
Wheat brain	17
Clover hay	35
Soybean meal (44% CP)	15
yellow corn	13
Molasses	4.0
Di-calcium phosphate	0.8
Limestone	0.5
DL-Methionine	0.1
Sodium chloride	0.3
Mixture (minerals + vitamins)^a^	0.3
**Chemical analysis (%)**	
Crude protein	18.2
Nitrogen free extract	56.97
Ash	9.18
Crude fibre	12.5
Ether extract	4.25
Digestible energy (kcal/ kg)	2650

^a^The vitamin and mineral premix/kg contained vitamin A, 6000 U; vitamin D_3_, 900 U; vitamin E, 40 mg; vitamin B_1_, vitamin K_3_, 2 mg; 2 mg; 4 mg; vitamin B_2_, vitamin B_6_, 2 mg; pantothenic acid, 10 mg; vitamin B_12_, 0.01 mg; Niacin, 50 mg; folic acid, 3 mg; choline, 250 mg; biotin, 0.05 mg; Fe, 50 mg; Cu, 5 mg; Mn, 85 mg; Co, 0.1 mg; Se, 0,1 mg; I, 0.2 mg and Zn, 50 mg

### Biological sampling

After 180 days of the trial, 10 female rabbits (10 days post-partum) were selected for laboratory analysis of various variables, including blood samples, tissue histology, and gene expression. The rabbits were euthanized according to Islamic guidelines [33], which do not require anesthesia for the animals, by severing the two jugular veins with a sharp knife. During the slaughtering, blood samples were collected in sterilized tubes. The tubes were left at room temperature for one hour and then centrifuged at 600 g for 15 min to collect serum. The serum was stored at -20°C for assessing blood biochemistry parameters. Samples of the ovaries, uterus, and liver were collected from the abdominal cavity and fixed in formalin for histological examination. Parts of the ovaries were stored at -80°C for gene expression analysis.

### Redox homeostasis assessment

Antioxidant and oxidative stress biomarkers were quantified using commercial ELISA kits. Superoxide dismutase (SOD) levels were determined using a sandwich enzyme immunoassay kit (Cat: ELK7383) from ELK Biotechnology Co., Ltd. (Denver, CO 80202, USA), following the protocol described by [34]. Total antioxidant capacity (TAC) and catalase (CAT) activities were measured using colorimetric kits (MBS2540515 and MBS1601538) from MyBioSource, Inc. (San Diego, USA), according to the methods outlined in [35] and [36] respectively. Glutathione peroxidase (GPx) and malondialdehyde (MDA) levels were determined with competitive ELISA kits (Cat: MBS754138 and MBS739495, respectively) from MyBioSource, Inc., following the methods described in [37], and [38] respectively.

### Immunoglobulins determinations

Serum levels of immunoglobulins (IgG and IgM) in rabbit does were determined at the end of the experimental period. IgG levels were measured using a SimpleStep ELISA® kit (Cat No. ab187400) for the quantitative determination of rabbit IgG. IgM levels were measured using a SimpleStep ELISA® kit (Cat No. ab190539) for the quantitative determination of rabbit IgM. Both assays were performed according to the manufacturer's instructions (abcam, USA). The assay sensitivity for IgG was 0.23 ng/mL with a detection range of 0.31—20 ng/mL. The assay sensitivity for IgM was 1.685 ng/mL with a detection range of 6.25—200 ng/mL.

### Reproductive hormonal assays

Serum levels of progesterone (PG), luteinizing hormone (LH), and follicle-stimulating hormone (FSH) in rabbit does were determined using commercially available ELISA kits designed specifically for rabbits. The specific kits for LH (ml027918), FSH (ml027868), and progesterone (ml028074) obtained from Mlbio Company (Shanghai, China). The assays were performed following the manufacturer's instructions, and the hormonal levels were determined by measuring the absorbance at 450 nm (OD values) using a microplate reader (Infinite F50, TECAN, Männedorf, Switzerland) with the blank set as the zero-reference.

#### Adipokines evaluation

Adipokine parameters, such as the levels of leptin, adiponectin, visfatin, and myokines like irisin, were assessed in the serum of rabbits exposed to heat stress conditions. The levels of irisin (Cat No. MBS2600900), adiponectin (Cat No. MBS083748), visfatin (Catalog No. MBS006687), and leptin (Catalog No. MBS704049) were determined using a commercial ELISA kit (MyBioSource, USA) employing the Quantitative Sandwich method with a spectrophotometric reader.

#### Reproductive performance

During the experimental period, we recorded key reproductive metrics, including conception rate, kindling rate, litter size at birth, and litter size at weaning. We also tracked the number of kits from birth to weaning. To confirm pregnancy, does were palpated 12 days post-mating. The conception rate (CR) was calculated using the formula: CR = (Number of delivered rabbit does / Number of pregnant rabbit does) × 100. Total litter size (TLS) at birth (12 h after kindling) and litter size at weaning (LSB) were recorded. Kits were individually numbered at birth, and their numbers were documented at 28 days.

#### Gene expression by Real-Time Quantitative PCR (RT-qPCR)

The ovarian tissues were immediately collected from four randomly chosen female rabbits at slaughter and immersed in liquid nitrogen (-196°C) before being transferred to the laboratory for RNA extraction. Total RNA was extracted using TRIzol (Invitrogen, Carlsbad, CA, USA) following the manufacturer's instructions. The quality and quantity of the extracted RNA were determined by measuring the 260:280 ratio using a NanoDrop 2000C instrument (Thermo Fisher Scientific, Wilmington, DE, USA). Real-time PCR was performed using ChamQ™ SYBR® qPCR Master Mix (Vazyme, China) on a QuantStudio® 5 Real-Time PCR System. The study investigated ferroptosis-related genes such as solute Carrier family 7 member 11 (*SLC7A11*), glutathione peroxidase 4 (*GPX4*), heat shock 70 (*HSP70*), and the DNA methylation gene DNA Methyltransferase 1 (*DNMT1*). The primer sequences are listed in Table 2. Each sample was examined three times, and the data was normalized to *GAPDH*. The relative gene expression level was calculated using the 2^^−ΔΔCt^ method [39].

**Table 2:** Primer information used in this study

Gene	Primer Sequence	Product Size (bp)	Accession Number
*DNMT1*	GAAGAACGTGGAGCTCTGCT AGGTCCTCGTAGGTGGAGTC	297	XM_051838133.1
*HSP70*	CCGGACTTTCCCTTCCCTTG TGACTATACACCGTCGCTACA	135	XM_051839570.1
*GPX4*	GTCTGAGTCGCCTGCTGAAG GCGGAGAACTCGTGCATAGA	124	XM_051840296.1
*SLC7A11*	ATCATCGGCGCAGGAATCTT GACAACCAGAGACATGCCCA	78	XM_008267436.3
*GAPDH*	CACCAGGGCTGCTTTTAACTCT CTTCCCGTTCTCAGCCTTGACC	145	NM_001082253.1

Solute carrier family 7 members 11 (*SLC7A11*), DNA methyltransferase 1 (*DNMT1*), glutathione peroxidase 4 (*GPX4*), heat shock 70 (*HSP70*)

#### Data statistical analysis

The Kolmogorov–Smirnov and Levene's tests were used to check the normal distribution of the data and the homogeneity of variances. In cases where normality assumptions were violated, we applied Tukey's multiple-range test and one-way ANOVA as our statistical methods. The data are presented as the mean ± standard error. A significant level of *P* < 0.05 was considered. The figures were generated using GraphPad Prism 8 with violin plots. PC-biplot and heatmap were generated using the ggplot2 and RcolorBrewer packages in R software.

## Results

### Characterization of BEON

The characterization of the synthesized BEON is illustrated in Fig. 1 (A, B, and C). The TEM image shows spherical and shapeless particles (Fig. 1A). The mean droplet diameter was measured to be 299.8 nm (Fig. 1B). The polydispersity index (PDI) was 0.166, indicating better stability (Fig. 1C).

### Climatic factors

The average ambient temperature (AT), relative humidity (RH), and temperature-humidity index (THI) throughout the entire experimental period were 31.68 ± 0.46, 66.55 ± 1.61 and 30.67 ± 0.21, respectively (Table 3). The THI recorded in this study suggests that the rabbit does experienced significant heat stress.

**Table 3:** The mean values of temperature–humidity index, relative humidity, and ambient temperature during the trial duration

Item	May–June	July- August	September- October	Overall period
AT (*◦*C)	31.57 ± 0.35	31.80 ± 0.62	31.67 ± 0.33	31.68 ± 0.46
RH (%)	63.67 ± 2.33	67.00 ± 1.53	69.00 ± 1.00	66.45 ± 1.61
THI	30.64 ± 0.33	31.05 ± 0.08	30.59 ± 0.19	30.7 ± 0.21

*AT *Ambient temperature, *RH *Relative humidity, *THI *Temperature–humidity index

### Effects on immunity and redox status

**Fig. 2:**
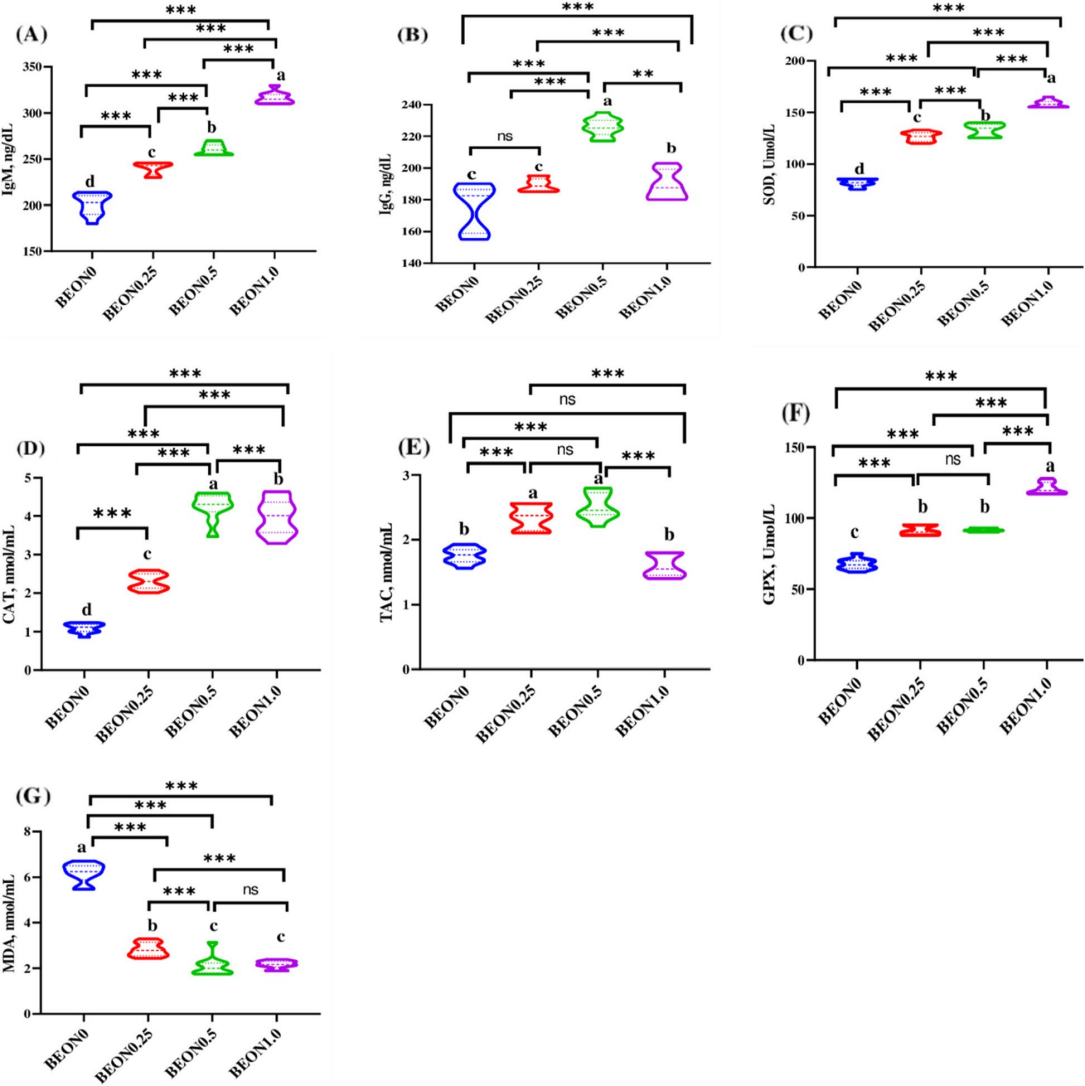
**A-G** Impacts of different levels of *boswellia* essential oil nano-formulation (BEON) on the redox homeostasis and immunity of heat-stressed rabbit does. Immunoglobulins M (IgM, **A**), G (IgG, **B**), superoxide dismutase (SOD, **C**), catalase (CAT, **D**), total antioxidant capacity (TAC, **E**), glutathione peroxidase (GPX, **F**), and malondialdehyde (MDA, **G**). Female rabbits were received basal diets and supplemented with 0 (BEON0), 0.25 (BEON0.25), 0.5 (BEON0.5) and 1.0 (BEON1.0) mL of boswellia essential oil nano-emulsion (BEON), kg diet during natural summer condition. Data presented as mean, *n* = 10 replicates ^a−d^ Different letters indicate significant differences between the groups (*P* < 0.05)

The effects of BEON on immunoglobulins (IgG and IgM) are presented in Fig. 2 (A-G). BEON dietary inclusion significantly increased the serum levels of IgM in a dose-dependent manner (Fig. 2A). IgG levels increased significantly (*P* < 0.05) in the BEON groups compared to the control group and the highest value was recorded with the BEON 0.5 group (Fig. 2B), which was significantly high compared to the other two BEON groups (0.25 and 1.0). The BEON treatment significantly increased the SOD levels (Fig. 2C) by 93.52%, 62.51%, and 54.6% with the addition of 0.25, 0.5, and 1 mL of BEON per kg of diet compared to the control group (*P* < 0.05). All studied BEON doses significantly increased (*P* < 0.05) CAT levels (Fig. 2D) compared to the control group, with increases of 111%, 290%, and 260% observed for 0.25, 0.5, and 1.0 mL BEON/kg diet, respectively. Among the BEON doses, the 0.5 mL/kg diet exhibited the highest effect on CAT levels, followed by 1.0 mL/kg and then 0.25 mL/kg. The TAC (Fig. 2E) value was significantly higher (*P* > 0.05) in BEON 0.25 and BEON 0.5 groups, with no significant differences observed between the high dose of BEON (1 mL) and the control group. The GPX (Fig. 2F) level showed a dose-dependent relationship with the BEON levels and reached its highest value at 1.0 mL of BEON per kg of diet compared to the control group (*P* < 0.001). Female rabbits given BEON in their diets had significantly lower levels of MDA (Fig. 2G) in their blood compared to the control group (*P* < 0.001). MDA levels decreased by 53.9%, 65.9%, and 65.5% with the addition of 0.25, 0.5, and 1.0 mL of BEON per kg of diet, respectively.

### Hormonal profile and adipokines

**Fig. 3:**
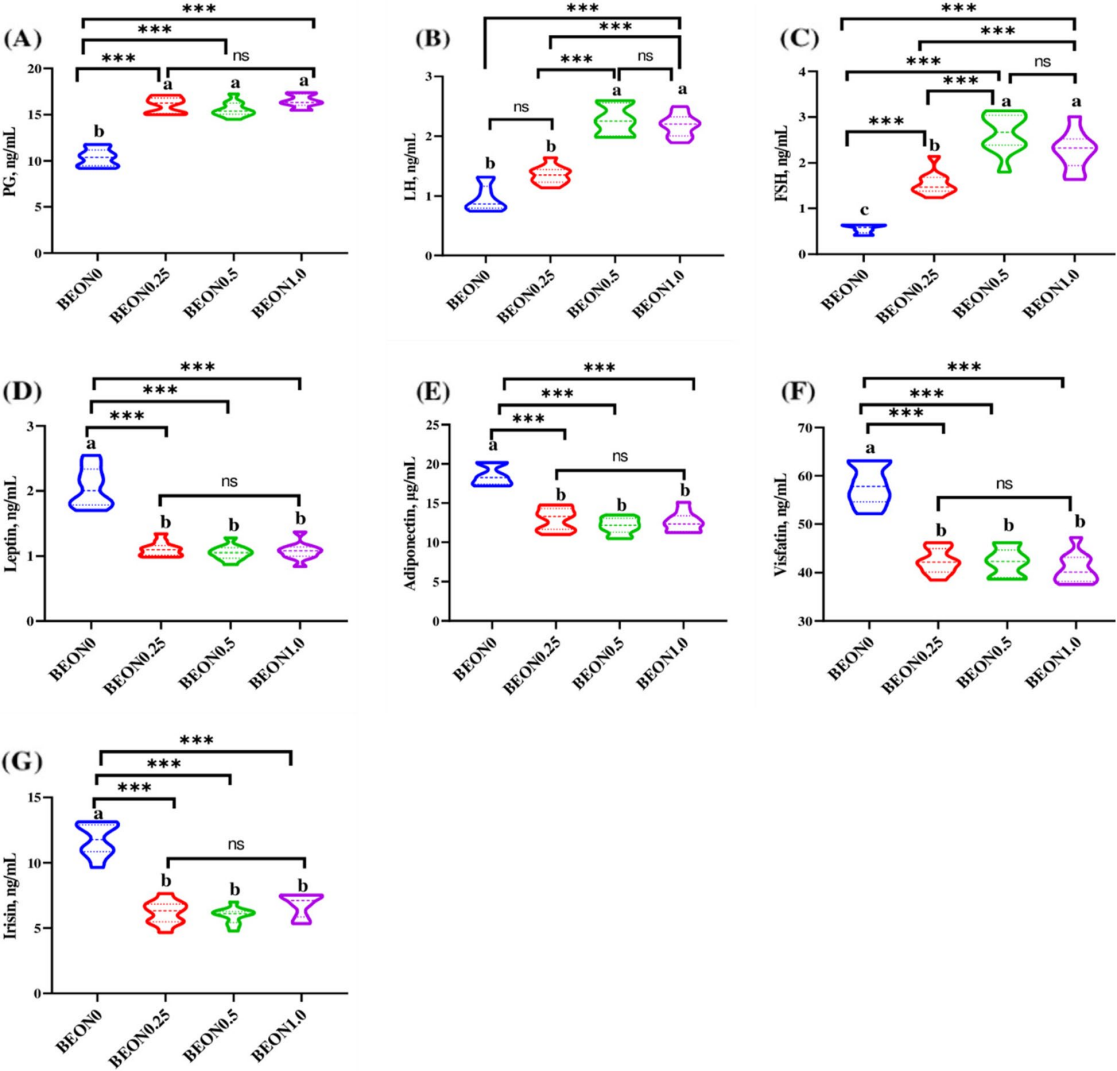
**A-G** Impacts of different levels of boswellia essential oil nano-formulation (BEON) on the hormonal profile and adipokines of heat-stressed rabbit does. Progesterone (PG, **A**), luteinizing hormone (LH, **B**), follicle stimulating hormone (FSH. **C**), leptin (**D**), adiponectin (**E**), vistatin (**F**) and irisin (**G**). Female rabbits were received basal diets and supplemented with 0 (BEON0), 0.25 (BEON0.25), 0.5 (BEON0.5) and 1.0 (BEON1.0) mL of boswellia essential oil nano-formulation (BEON), kg diet during natural summer condition. Data presented as mean, *n* = 10 replicates ^a−c^ Different letters indicate significant differences between the groups (*P* < 0.05)

Impacts of BEON dietary supplementation on the hormonal profile of stressed female rabbits are clarified in Fig. 3 (A-G). Female rabbits fed diets supplemented with different doses of BEON showed significantly higher PG (Fig. 3A) levels compared to the control group (*P* < 0.001). Addition of BEON to HS does increase serum LH compared to the untreated group but these increases were significant (*P* < 0.05) with 0.5 and 1 mL of BEON only (Fig. 3B). Feeding heat-stressed female rabbits with a diet supplemented with 0.25, 0.5, or 1.0 mL BEON/kg diet significantly increased serum FSH hormone (Fig. 3C) compared with the untreated group (*P* < 0.001) and the two highest levels of BEON had a significantly higher effect on increasing the FSH hormone from its low level. The serum activities of leptin (Fig. 3D) and visfatin (Fig. 3F) gradually and significantly decreased in rabbits fed diets containing BEON (0.25–1 mL/kg diet) and this decrease was BEON dose dependent manner. Adiponectin (Fig. 3E) and Irisin (Fig. 3G) myokine levels were significantly decreased in treated groups during heat stress conditions compared to untreated group and the 0.5 BEON level showed a higher reduction effect than the other two tested levels. The reduction was 47.1- 29.1%, 49.5—34.2%, and 41.3—31.7% for 0.25, 0.5, and 1 mL of BEON respectively.

### Reproductive features

All supplemented groups exhibited higher conception and kindling rates compared to the control groups, with the most favorable outcomes observed in the BEON 0.5 and BEON 1.0 groups (*P* < 0.05; Table 4). Litter size at birth and weaning was greater in all BEON groups in comparison to the control group. The bunnies mortality rate was higher in the control group than in the other tested groups. The BEON groups (0.5 and 1.0 mL/kg diet) had fewer deceased bunnies compared to the BEON 0.25 group.

**Table 4:** Impacts of different levels of boswellia essential oil nano-formulation (BEON) on the reproductive performance of heat-stressed rabbit does

Item	Experimental groups^*^				*P* value
	**BEON0**	**BEON0.25**	**BEON0.5**	**BEON1.0**	
No. of females	30	30	30	30	–
Conception rate (%)	19 (63.3%)	22 (73.3%)	25 (83.3%)	25 (83.3%)	–
Kindling rate (%)	17 (56.67%)	22 (73.3%)	25 (83.3%)	25 (83.3%)	
Litter size at birth (n)	5.00 ± 0.12^b^	6.25 ± 0.15^a^	6.25 ± 0.14^a^	6.25 ± 0.13^a^	0.073
Litter size at weaning (n)	3.50 ± 0.18^b^	5.50 ± 0.12^a^	5.75 ± 0.17^a^	5.75 ± 0.18^a^	0.000
No. of dead bunnies (birth to waning)	35%	13.6%	8.6%	8.6%	–

^a^Female rabbits were received basal diets and supplemented with 0 (BEON0), 0.25 (BEON0.25), 0.5 (BEON0.5) and 1.0 (BEON1.0) mL of boswellia essential oil nano-emulsion (BEON), kg diet during natural summer condition. Data presented as mean ± SE, *n* = 10 replicates

^a^^−^^b^ Different letters in each row indicate significant differences between the groups (*P* < 0.05)

### Ferroptosis-related genes and epigenetic changes

**Fig. 4:**
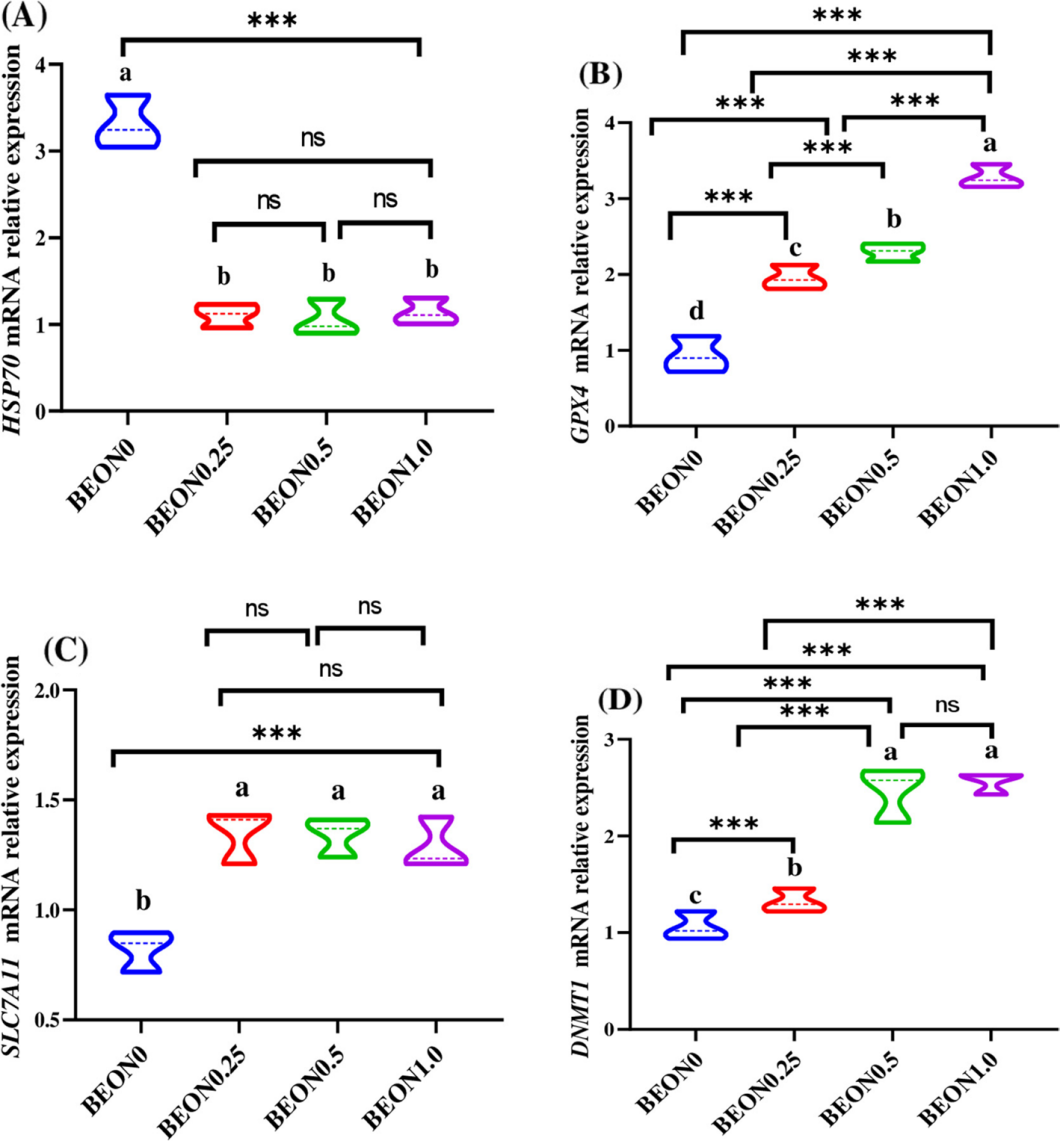
**A-D** Impacts of different levels of boswellia essential oil nano-formulation (BEON) on ferroptosis-related genes such as *HSP70* (**A**), *GPX4* (**B**) and *SLC7A11* (**C**) and epigenetic mRNA expression of *DNMT1* (**D**) in ovarian tissues of heat-stressed rabbit does. Rabbits were fed diets with 0 (BEON0), 0.25 (BEON0.25), 0.5 (BEON0.5) and 1.0 (BEON1.0) of boswellia essential oil nano-formulation (BEON)/kg diet. Data presented as mean, *n* = 10 replicates^. a−d^ Different letters indicate significant differences between the groups (*P* < 0.05)

As depicted in Fig. 4, dietary supplementation with BEON significantly downregulated the expression of ferroptosis-related genes such as *HSP70* (Fig. 4A), while increasing the expression of *GPX4* (Fig. 4B) and *SLC7A11* (Fig. 4C) in ovarian tissues (*P* < 0.001). The epigenetic mRNA expression of *DNMT1* (Fig. 4D) was significantly increased in a dose-dependent manner with increasing levels of BEON in rabbit diets. The mRNA expression of *HSP70* was lower in female rabbits fed diets containing 0.25–1.0 mL of BEON compared to the control group (*P* < 0.05). Additionally, the mRNA expression of *GPX4* and *SLC7A11* increased in a dose-dependent manner with higher levels of BEON in rabbit diets.

### Organs histology

**Fig. 5:**
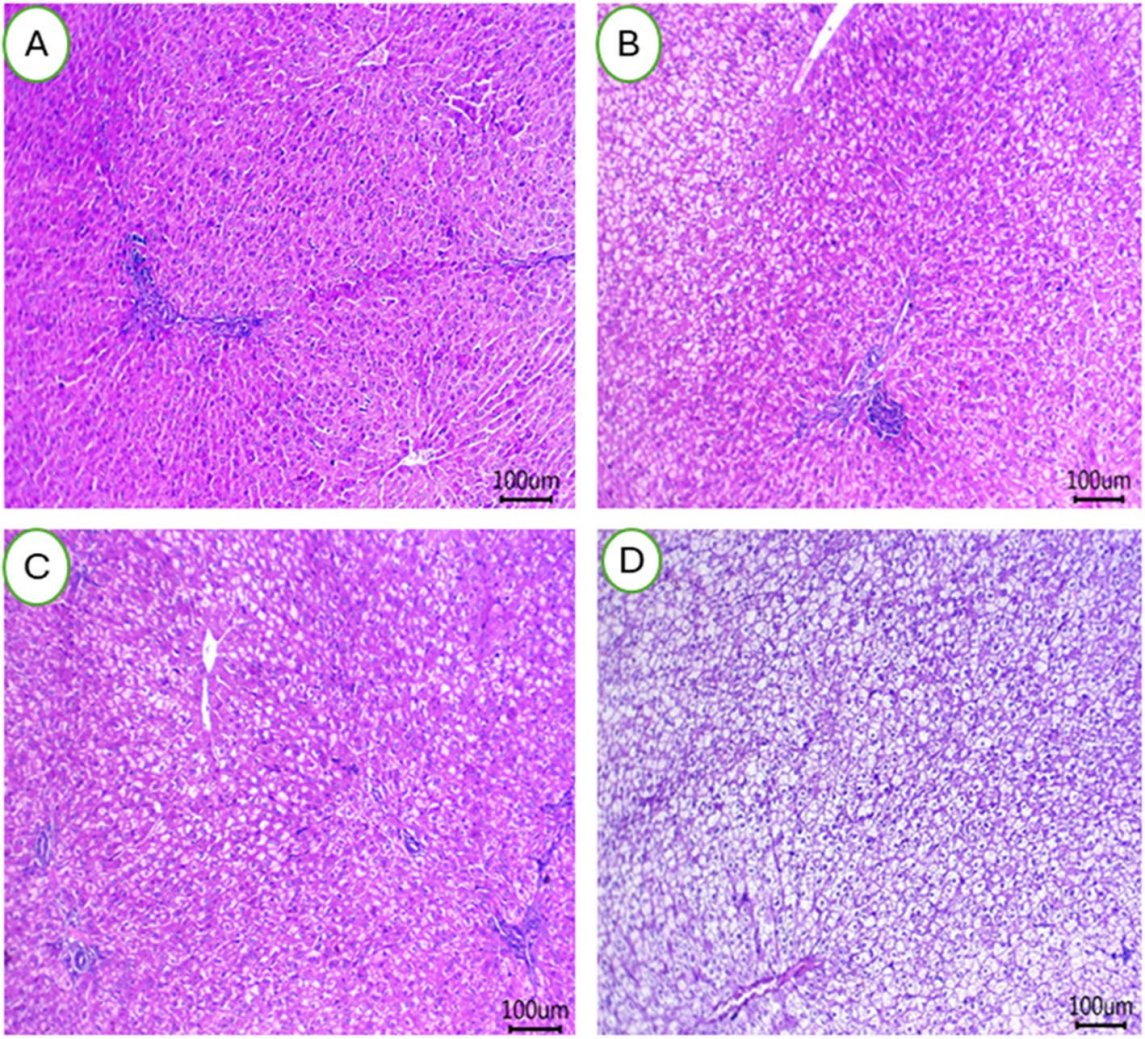
**A-D** The hepatic tissues of female rabbits were examined after being fed a basal diet (CON group; (**A**) or a diet supplemented with BEON at 0.25 (**B**), 0.5 (**C**), and 1 (**D**) mL/kg during summer conditions

**Fig. 6:**
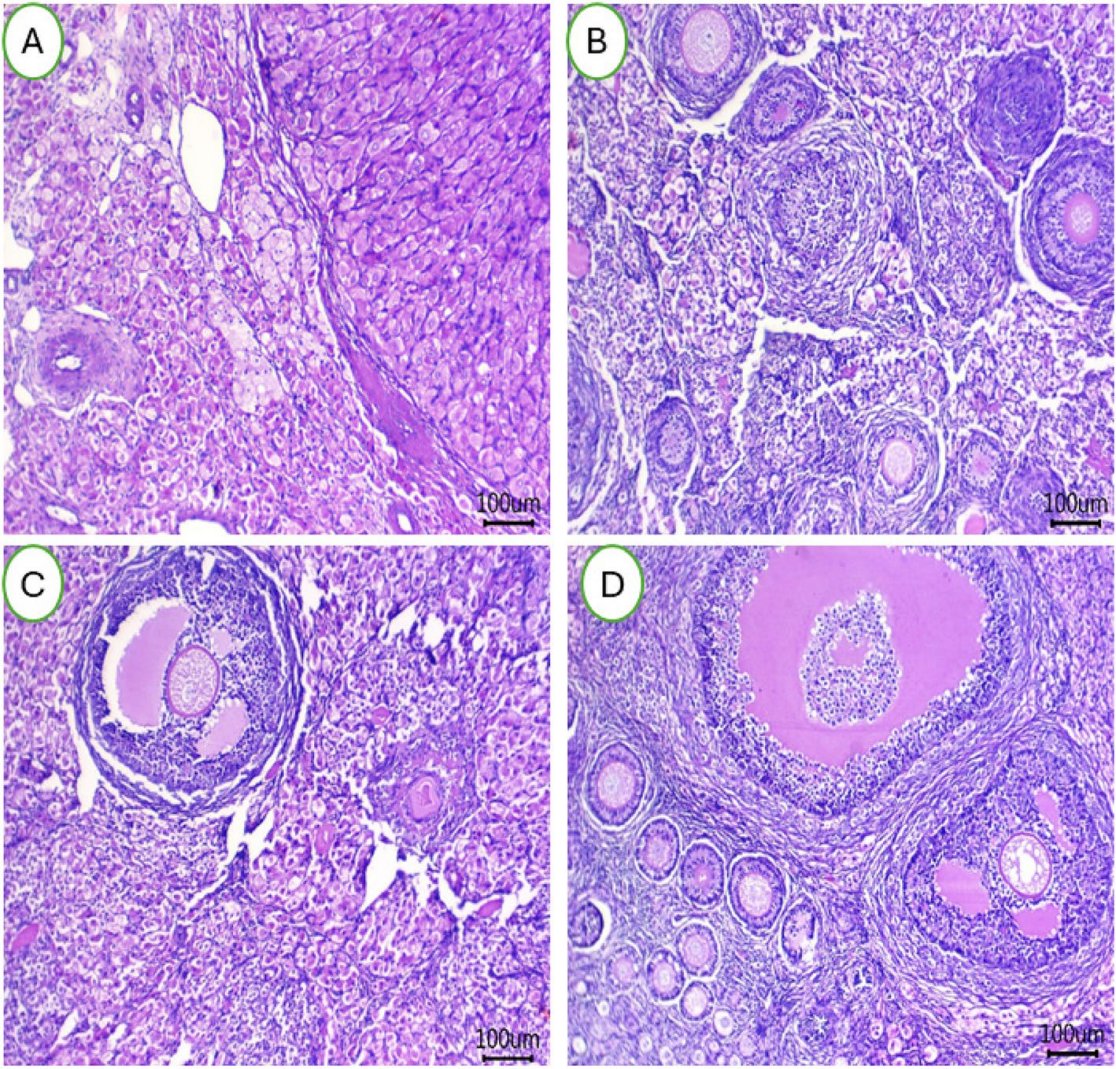
**A-D** The ovarian tissues of female rabbits were examined after being fed a basal diet (CON group; (**A**) or a diet supplemented with BEON at 0.25 (**B**), 0.5 (**C**), and 1 (**D**) mL/kg during summer conditions

The impact of HS on liver tissues of female rabbits and potential use of BEON to mitigate these negative effects are presented in Fig. 5 (A-D). Rearing female rabbits under HS conditions and feeding them basal diets resulted in mild congestion of hepatic blood vessels with small perivascular lymphocytic infiltrations (Fig. 5A). Some inflammatory cells were also observed. In contrast, female rabbits fed diets supplemented with different levels of 0.25 mL (Fig. 5B), 0.5 mL (Fig. 5C), and 1 mL (Fig. 5D) BEON/kg diet showed normal hepatic cords, hepatic sinusoids, and central veins in their hepatic tissues. Female rabbits exposed to heat stress showed severe impacts on ovarian tissues, and BEON can eliminate this effect (Fig. 6A-D). The number of follicles at each stage did not differ significantly, but their qualitative was affected, whereas, HS may increase the number of vacuolated follicles, especially primordial follicles (Fig. 6A). The HS group exhibited fewer atretic and Graafian follicles, with dilated antrum and either absent or degenerated ova (Fig. 4A). In the treated groups, it can be seen that healthy follicles increased in number with the BEON0.25 group (Fig. 6B), while the BEON0.5 (Fig. 6C) and BEON1.0 (Fig. 6D) groups had a greater number of Graafian follicles and fewer atretic follicles, indicating healthy ovarian tissues affecting reproductive efficiency.

**Fig. 7:**
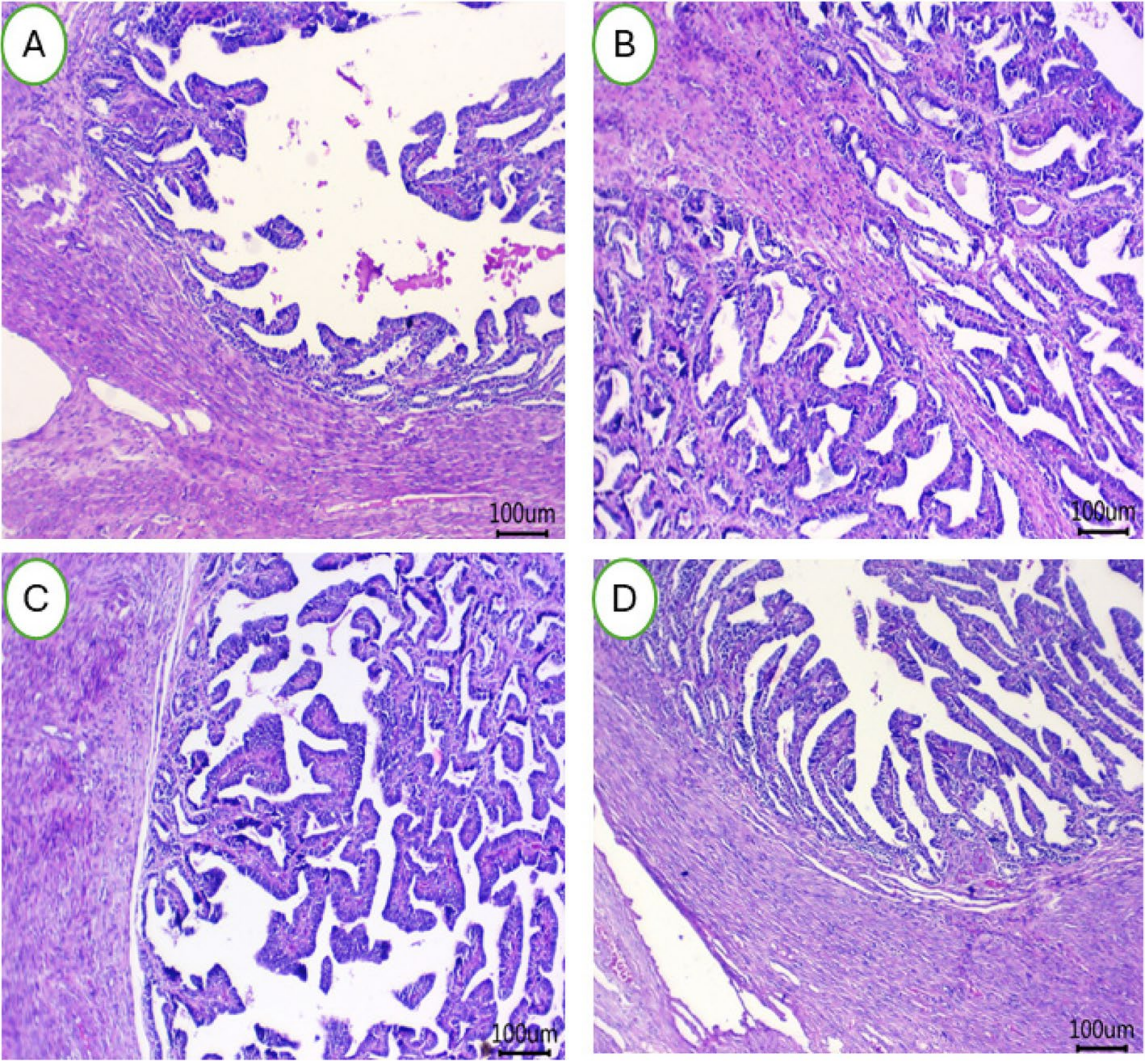
**A-D** The uterine horns tissues (right) of female rabbits were examined after being fed a basal diet (CON group; (**A**) or a diet supplemented with BEON at 0.25 (**B**), 0.5 (**C**), and 1 (**D**) mL/kg during summer conditions

Figure 7 (A-D) illustrates the histological impact of HS on uterine horns in female rabbits and demonstrates the ameliorative effects of BEON treatment. The HS group exhibited signs of inflammation, including the presence of inflammatory cells and damage to the uterine glands, potentially compromising nutrient supply to pre-implantation zygotes (Fig. 7A). In contrast, rabbits treated with 0.25 mL (Fig. 7B), 0.5 mL (Fig. 7C), and 1.0 mL (Fig. 7D) BEON/kg diet displayed normal endometrial architecture with intact epithelial linings. Notably, the 0.5 mL and 1.0 mL BEON/kg diet groups exhibited an absence of perivascular lymphocytic infiltrations.

### Principal component analysis (PCA)

**Fig. 8:**
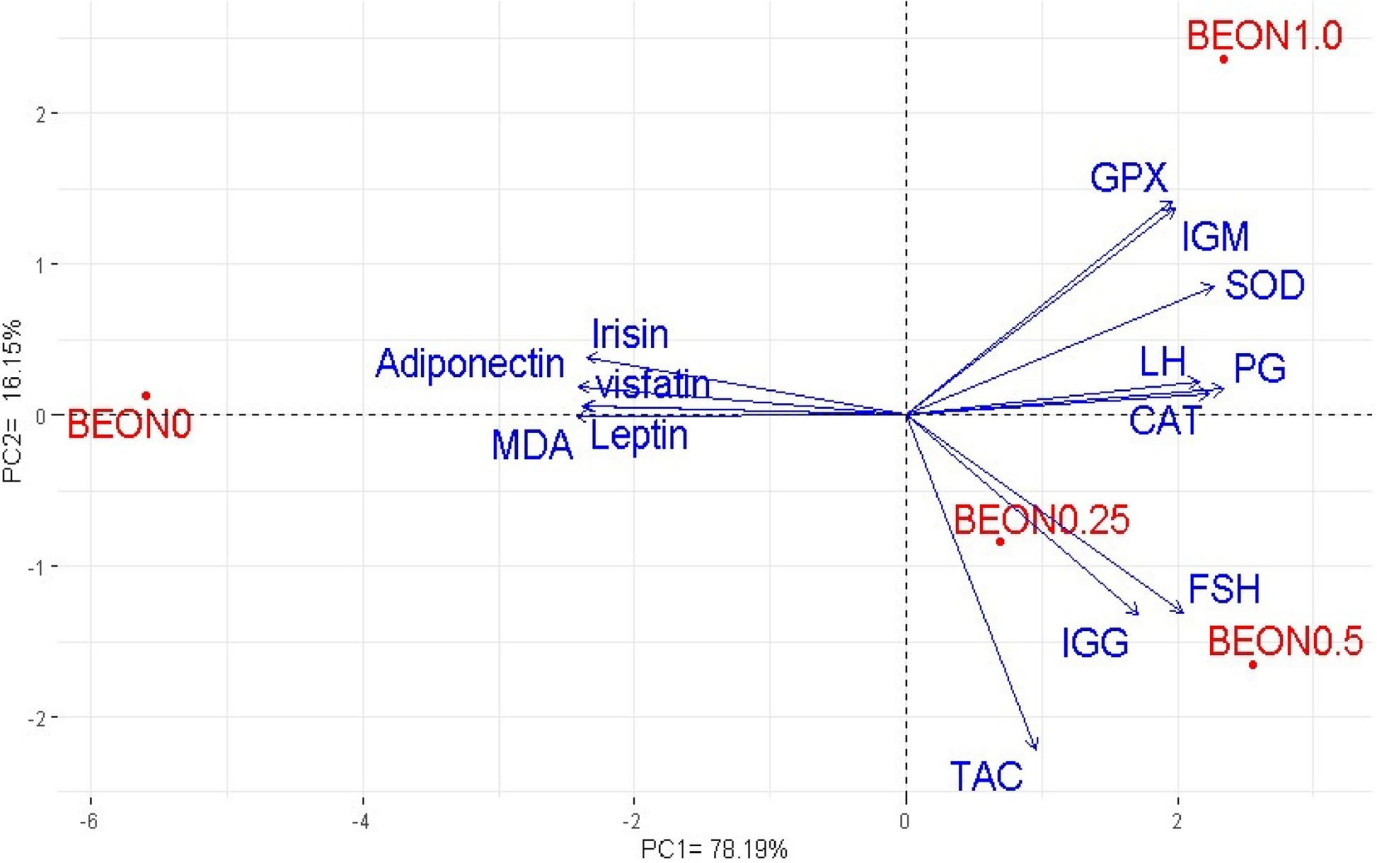
Principal component biplot (**A**) for the dietary supplementation of BOEN on some investigated variables sin female stressed rabbits. Female rabbits were received basal diets and supplemented with 0 (BEON0), 0.25 (BEON0.25), 0.5 (BEON0.5) and 1.0 (BEON1.0) mL of boswellia essential oil nano-emulsion (BEON), kg diet during natural summer condition

PCA was applied to investigate the intricate interplay between various therapies and key evaluated parameters, including immunity, antioxidant status, reproductive hormone levels, and adipokine concentrations in female rabbits exposed to hot climatic conditions (Fig. 8).

The first two PCAs explained the maximum variability of around 94.34% (78.19% by PC1 and 16.15% by PC2). PC1 was linked to BEON supplementation and factors such as immune function (IgG and IgM), antioxidants (CTA, SOD, TAC, and GPX), and reproductive hormones (PG, LH, and FSH) on the positive side. In contrast, the HS group was positioned on the negative side of PC1, along with MDA and adipokine hormones (leptin, adiponectin, visfatin, and irisin). All mentioned biologically variables, except MDA and adipokine hormones, showed a strong positive correlation with BEON dietary inclusion.

### Correlation study using heatmap clustering

**Fig. 9:**
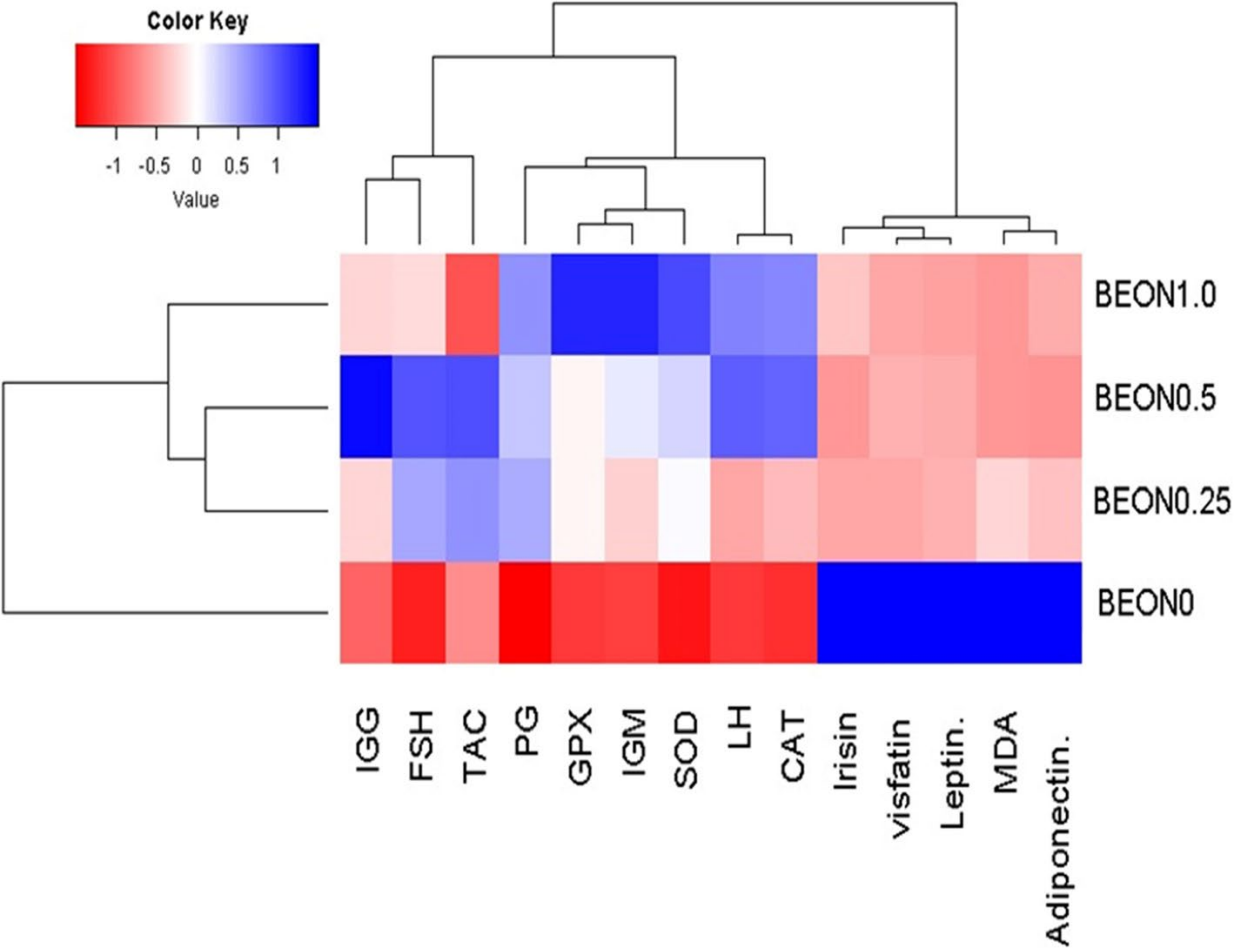
The heatmap and hierarchical clustering analysis for the dietary supplementation of BOEN on some investigated variables sin female stressed rabbits. Female rabbits were received basal diets and supplemented with 0 (BEON0), 0.25 (BEON0.25), 0.5 (BEON0.5) and 1.0 (BEON1.0) mL of boswellia essential oil nano-formulation (BEON), kg diet during natural summer condition

Heatmap clustering was performed based on the studied physiological parameters and the inclusion of BEON in the diet under HS conditions (Fig. 9). BEON supplementation emerged as the primary factor distinguishing the main clusters. The HS group exhibited the lowest values (represented by red), while the dietary inclusion of BEON demonstrated the highest values for most of the studied parameters (blue color). BEON supplementation in the diets of female rabbits showed a positive correlation with several investigated traits related to immunity, antioxidants, and reproductive hormones (represented by red), whereas a negative correlation was observed with MDA and adipokines (blue color).

## Discussion

This study demonstrates that heat stress (HS) negatively impacts reproductive performance in rabbits. These adverse effects are manifested by an imbalanced redox status, upregulation of ferroptosis-related gene expression, suppressed immune function, and dysregulated reproductive hormone and adipokines, levels. Dietary supplementation with a nano-emulsion of boswellia essential oil (BEON) was found to enhance reproductive performance in heat-stressed rabbits. These beneficial effects are likely attributed to BEON's ability to modulate ferroptosis and adipokine levels. Notably, BEON contains bioactive compounds such as p-Cymene, α-Pinene, d-Limonene, α-Thujene, α-Phellandrene, Terpinolene, delta-3-carene, myrcene, trans-verbenol, Sabinene, lupeolic acids, and boswellic acids. These compounds exhibit antimicrobial, antibacterial, anti-inflammatory, and anti-apoptotic properties and possess the potential to regulate ferroptosis pathways (Table 5). For instance, p-Cymene, a compound found in oils of, has been shown to have antioxidant properties, which can help reduce oxidative stress, suppress TNF-α/NF-κB, and enhance Nrf2/HO-1 expression in immobilized rats [40].

**Table 5:** The main bioactive compound presented in the *Boswellia serrata* resin oil based on the literature

Compound	2D structure	Main biological activity	Reference
P-Cymene		- Antioxidant - Hepatoprotective	[22, 40]
α-Pinene		- Antioxidants - Ferroptosis mediator	[22]
d-Limonene		- Autophagy regulator - Anti-apoptosis	[41]
α-Thujene		- Antibacterial activity	[22]
α-Phellandrene		- Promoting vascular endothelial growth factor - Stimulating cAMP	[28]
Terpinolene		- Anti-inflammatory effects	[42]
delta-3-Carene		- Antioxidant activity	[43]
Myrcene		- Antioxidant activity	[44]
*trans*-verbenol		- Antioxidant activity	[44]
Sabinene		- Antimicrobial activity	[45]
Lupeolic acids		- Anti-inflammatory effects	[25, 46]
Boswellic acids		- Antiproliferative, - Antioxidant and antimicrobial - Anti-inflammatory	[25, 47]

Heat stress can induce oxidative stress and lipid peroxidation, negatively impacting animal health and productivity. In this study, heat-stressed female rabbits exhibited decreased levels of immune markers (IgG and IgM) and antioxidant enzymes (SOD, CAT, GPX, and TAC), while simultaneously experiencing an increase in MDA levels, a marker of lipid peroxidation. Moreover, dietary supplementation with BEON (at 0.5 or 1 mL/kg) in heat-stressed rabbits modulated both immune function and antioxidant status. These findings are consistent with previous research [4, 48], that demonstrated the potential of herbal supplements and their oils to enhance immune function and antioxidant status in rabbits subjected to humid tropical conditions. Specifically, previous studies have demonstrated that *Boswellia serrata* enhances antioxidant enzyme activity, such as GSH and SOD, in both rabbits [19] and broilers [18, 20, 49]. GSH plays a crucial role in protecting cells from oxidative stress. SOD is a crucial antioxidant enzyme that catalyzes the conversion of superoxide radicals (O_2_-) into hydrogen peroxide (H_2_O_2_) [9]. Superoxide radicals are highly reactive molecules generated during cellular metabolism, and excessive levels can contribute to OS [50]. Consistent with these findings, our study demonstrated that supplementing heat-stressed rabbits with Boswellia serrata resin significantly reduced MDA levels compared to the control group. This finding supports the theory that *Boswellia serrata*'s antioxidant properties, primarily attributed to its high boswellic acid content (25–30%), can offer protection against OS [47]. Elevated ambient temperatures can lead to increased OS and lipid accumulation, rendering rabbits more susceptible to lipid peroxidation. Consistent with this observation, [51], reported that HS promotes increased adipocyte triacylglycerols (TAG) storage, potentially through the upregulation of genes involved in fatty acid uptake and TAG synthesis. Our findings align with those of [48] which demonstrated that herbal supplementation can enhance the oxidative stability of female rabbits by mitigating MDA and augmenting antioxidant activities under HS conditions. BEON contains α-Phellandrene, a compound known to activate cAMP signaling [28], potentially leading to the upregulation of vascular endothelial growth factor, a key mediator of heat dissipation.

HS weakens the immune system by increasing OS and suppressing immunoglobulin synthesis. Our study indicates that dietary supplementation with BEON resulted in a notable increase in both IgM and IgG levels, aligning with previous research conducted in rabbits [19] and broilers [18, 20] under normal conditions. *Boswellia serrata* may directly modulate the immune system by stimulating the activity of immune cells such as lymphocytes (B cells and T cells), which are responsible for producing antibodies like IgM and IgG [20]. Some authors suggest that the anti-inflammatory and antioxidant activities of *Boswellia serrata* may contribute to increased IgM and IgG levels by neutralizing free radicals and reducing oxidative damage [21, 52]. Further research is necessary to fully elucidate the specific pathways involved.

Reproductive hormones such as PG, LH, and FSH play crucial roles in the reproductive process. PG supports pregnancy, LH triggers ovulation, and FSH stimulates follicle development. HS can disrupt reproductive function by adversely affecting the hypothalamic-pituitary–gonadal axis, leading to a decrease in the synthesis of these crucial reproductive hormones in rabbits [3, 4]. Previous studies have shown that HS significantly reduces the levels of P4, LH, and FSH [10, 11]. Our research also found that HS decreased the levels of these hormones, while BEON administration significantly improved their circulating levels. These results are consistent with previous studies in rabbits [2, 4, 6], that demonstrated the positive effects of dietary herbs, such as moringa, thyme, and herbal mixtures, on reproductive hormone production. The antioxidant properties of these herbs may contribute to their ability to effectively mitigate OS [4, 29], which can adversely impact reproductive hormone function.

Beyond lipid storage, adipose tissues function as metabolic and endocrine organs, secreting hormone-like mediators known as adipokines. Adipokines, including adiponectin and leptin, play pivotal roles in regulating diverse physiological processes, such as glucose and lipid metabolism, energy balance, reproductive function, redox homeostasis, and immune and inflammatory responses [53]. HS can adversely affect reproductive function, potentially through disruptions to the adipokine-reproductive axis. This study observed significant increases in circulating leptin, adiponectin, visfatin, and irisin levels in stressed female rabbits. Notably, BEON supplementation effectively lowered the circulating levels of all examined adipokines in stressed rabbits. Adiponectin, a key hormone synthesized by adipose tissue, exerts potential influence on reproductive processes [54]. Leptin, primarily secreted by fat cells, plays a pivotal role in regulating body weight and energy homeostasis. Irisin recognized as a crucial neurotransmitter, mediates glucose and lipid metabolism [53]. Consistent with our findings, serum adiponectin levels were observed to be higher in Simmental cows during summer compared to autumn [55].

Our results corroborate previous research demonstrating a significant increase in circulating leptin levels in various species, including cows [56], goats [57] and broiler [54]. This study further supports the notion that elevated leptin levels in heat-stressed rabbits [6, 48], likely signify disruptions in energy homeostasis. These disruptions can encompass altered appetite, reduced thyroid hormone production, and increased water intake. This observation aligns with studies in growing rabbits [6, 48], that also reported elevated adiponectin levels under HS conditions.

Heat stimulation can elevate blood irisin levels in humans [43]. While elevated circulating irisin levels are often associated with muscle damage and increased OS [58], high levels may also indicate an uncontrolled secretion of this hormone triggered by abiotic stressors such as HS. Despite these potential drawbacks, irisin has recently demonstrated significant promise in therapeutic applications, including cardioprotection and stress mitigation [59]. Furthermore, [54] demonstrated that elevated adiponectin levels in stressed broiler chickens can be mitigated by incorporating certain herbs into their diets. In line with our data, studies have demonstrated that incorporating herb-enriched diets into the feeding regimen of stressed rabbits can contribute to a reduction in elevated leptin and adiponectin levels [2, 6, 48]. The findings suggest that BEON effectively modulates the increase in adiponectin, irisin, and leptin levels induced by HS. This indicates that dietary BEON can regulate circulating irisin levels, supporting animal health by enhancing mitochondrial function and reducing OS. Visfatin, an adipocytokine with various properties, has been linked to reproductive performance in pigs and conditions like brain damage [60], and polycystic ovary syndrome in women [61]. Elevated visfatin levels may contribute to infertility in animals. Our study observed a significant increase in visfatin during HS, correlating with reduced reproductive performance in rabbits. Treatment with BEON, rich in antioxidant molecules such as α-Pinene, boswellic acids, p-Cymene, and d-Limonene [19, 27, 52], resulted in a significant decrease in visfatin levels, consequently enhancing reproductive health in rabbit. In contrast, [55] clarified that high environmental temperatures decreased leptin levels in Simmental cows compared to winter conditions.

Heat stress (HS) can disrupt molecular chaperones in the cellular system, leading to various cellular processes, including ferroptosis [8, 62]. Ferroptosis, a regulated form of cell death characterized by iron-mediated accumulation of lipid peroxides, provides a novel avenue for exploring the intersection of cellular metabolism, oxidative stress, and disease pathology. HSP70 is a protein that responds to cellular stress, particularly oxidative stress, and is highly expressed in tissues exposed to HS [54]. Our data shows that HS significantly increased the expression of *HSP70* and *SLC7A11*, while significantly reducing the expression of *GPX4* in the ovarian tissue of rabbits. Feeding rabbits with BEON at various levels supported the expression of *GPX4* in ovarian tissues. This elevation in BEON administration aligns with the previously reported increase in antioxidant enzymes in the blood serum. Previous studies have demonstrated that certain natural compounds, such as essential oils, can effectively reduce *HSP70* expression in stressed rabbit [2, 48], and broiler [54] and rabbit bucks [6]. Reduced mRNA levels of HSP70 may indicate a decrease in cellular abiotic stress, as evidenced by the antioxidant activity of boswellic acids, p-Cymene, α-Pinene, d-Limonene, α-Thujene, and α-Phellandrene present in BEON (Table 5).

BEON treatment can lower *HSP70* and *SLC7A11* expression, indicating reduced cellular stress. While elevated *HSP70* can be a protective response, decreased levels may indicate restored cellular homeostasis. *SLC7A11* plays a well-established role in protecting cells from oxidative stress-induced cell death, such as ferroptosis, and is overexpressed in many human cancers [63]. *SLC7A11*-mediated cystine transport is crucial for suppressing ferroptosis, a form of iron-dependent, regulated cell death triggered by unchecked lipid peroxidation in cellular membranes [63]. BEON improved HS-induced ferroptosis in ovarian cells by activating the *SLC7A11/GPX4* pathway, while *HSP70* overexpression effectively negated the protective effect of BEON. This hypothesis was also supported by a study in mice by [62]. Based on these results, we suggest that natural nano-emulsion product-derived ferroptosis regulators emerge as a potential strategy for treating HS in the livestock industry [14].

Heat stress (HS) can induce epigenetic alterations by influencing DNA methylation. DNA methyltransferase 1 (*DNMT1*) is a key enzyme responsible for DNA methylation, a critical process regulating gene expression. This study found that HS significantly reduced *DNMT1* levels in rabbit ovarian tissues. Conversely, supplementation with BEON upregulated *DNMT1* expression, suggesting a role in maintaining proper gene regulation. These findings corroborate previous research demonstrating DNA hypomethylation in cattle subjected to acute heat stress [64]. Notably, mRNA expression of *DNMT1*, essential for oocyte and embryo development, was significantly lower in bovine oocytes exposed to HS [65, 66]. Pavani et al. [66] further observed significant downregulation of *DNMT1* in embryos produced during warmer months, highlighting a potential link between heat stress and impaired developmental competence. Targeting DNA methylation represents a promising strategic approach for improving livestock resilience to climate change. To further investigate the protective effects of BEON, we conducted histological examinations of the liver, ovaries, and uterine horns.

Additionally, HS significantly compromises reproductive capacity in rabbits, hindering farm productivity. This study demonstrates that supplementing rabbit diets with 0.5 or 1 mL of BEON (83.3% *vs*. 63.3%) significantly enhanced reproductive performance compared to the control group. Specifically, we observed a notable increase in conception rates (83.3% vs. 63.3% in controls), kindling rate, litter size at birth, and weaning rate, while significantly reducing the number of dead bunnies (*P* < 0.05). These findings align with previous research [4], showing that thyme oil positively impacts reproductive health in rabbits under elevated temperatures. The improved reproductive outcomes may be attributed to BEON's ability to modulate the immune system, potentially through the upregulation of anti-inflammatory cytokines such as lysozyme, complement C, and IL-10, as observed in broiler chickens supplemented with varying levels of BEON [20].

The liver is a highly sensitive organ to elevated ambient temperatures [67]. In animal models, HS increases portal venous blood flow, leading to hepatic hypoxia and cellular damage [67]. Histopathological studies in heat-stressed rabbits have shown evidence of hepatocellular fatty degeneration and pronounced cellular damage [67]. This sensitivity is particularly evident in rabbits [11]. HS also negatively impacts reproductive function in rabbits. In heat-stressed animals, ovarian tissue exhibits reduced numbers of atretic and Graafian follicles, with dilated antra and often absent or degenerated ova [10, 11]. This suggests a decline in reproductive capacity during hot climates. While HS adversely affects the ovaries, it appears to have less impact on the uterine structure [10]. All treated groups in the study showed a normal endometrial layer with healthy lining epithelia in the uterine horns. This may be attributed to the potential protective effects of BEON, given its antioxidant, immunomodulatory and anti-inflammatory properties [18, 20, 27]. Previous studies have shown that BEON can increase antioxidant levels in the blood and improve immune function. In the heat-stressed group, the uterus displayed sub-endometrial edema, characterized by inactive epithelia and leukocytic infiltration. However, the treatments effectively mitigated these HS-induced alterations by promoting uterine gland secretion and reducing inflammation [10, 11].

PCA and heatmap clustering were used to investigate the relationships between BEON inclusion and evaluated parameters, a methodology utilized in published reports [68, 69]. PCA effectively reveals the overall structure and major sources of variation within the data, identifying clusters of rabbits with similar physiological profiles. The PCA biplot and heatmap analysis showed positive associations between BEON supplementation and immune function, antioxidant capacity, and reproductive hormone levels, while indicating negative associations with MDA and adipokines in rabbits experiencing HS. These findings suggest that BEON supplementation may serve as a promising nutritional intervention to enhance reproductive performance and climate change resilience in female animals. This study presents several limitations. Nevertheless, our findings indicate that BEON may enhance heat resistance in rabbits by reducing ferroptosis in ovarian tissue. Further research is necessary, including gene expression analysis and protein expression assessments via Western blotting, to validate these results. This study focused solely on the effect of BEON on DNMT1 in ovarian tissues. Due to resource limitations, the influence of BEON on other key DNA methylation enzymes, such as *DNMT3A* and *DNMT3B*, was not investigated. Additionally, limitations of this study include a relatively small sample size due to resource constraints and a lack of data on the fertility capacity of the male rabbits used for artificial insemination. Future studies should include Western blotting and immune reactivity analyses to further validate these findings.

## Conclusion

This study investigates the use of nanotechnology to enhance heat resistance in female rabbits. The reuslts indicates that incorporating a nano-emulsion of boswellia serrata resin oil (BEON, 0.5–1.0 mL/kg diet) into the rabbits' diet shown improvements in reproductive efficiency, immune function, and antioxidant status. BEON was shown to decrease ferroptosis and regulate adipokine hormones. Moreover, it affected DNA methylation in ovarian tissues, suggesting a possible epigenetic mechanism. These results underscore the potential of nanotechnology in alleviating the adverse effects of heat stress on rabbit reproduction and promoting sustainable rabbit farming practices.

## Data Availability

The datasets used and/or analysed during the current study available from the corresponding author on reasonable request.
